# Development of a machine learning-based predictive model for osteoporosis risk and its application in clinical decision support

**DOI:** 10.3389/fmed.2025.1680731

**Published:** 2025-11-13

**Authors:** Zichen Shao, Jianfeng Wu, Qinqin Deng, Ling Cheng, Xin Huang, Weikang Sun, Weidong Liang, Huanan Li

**Affiliations:** 1 Jiangxi University of Chinese Medicine, Nanchang, Jiangxi, China; 2 Affiliated Hospital of Jiangxi University of Chinese Medicine, Nanchang, Jiangxi, China

**Keywords:** osteoporosis, machine learning, LASSO regression, Random Forest, SHAP, clinical decision support

## Abstract

**Objective:**

This study was aimed at developing an interpretable machine learning model for predicting osteoporosis (OP) risk using real-world clinical data, and at establishing a web-based visualization tool for assisting clinical decision-making.

**Methods:**

A total of 5,328 individuals from the Affiliated Hospital of Jiangxi University of Chinese Medicine (2015–2024) were included. Multidimensional data, including demographic characteristics, anthropometric measures, lumbar spine bone mineral density (L1–L4), and more than 90 blood biochemical and inflammatory markers, were collected. Key variables were identified using univariate analysis followed by least absolute shrinkage and selection operator (LASSO) regression. Five machine learning algorithms—Decision Tree, Random Forest, XGBoost, CatBoost, and Multi-Layer Perceptron (MLP)—were developed and compared. SHapley Additive exPlanations (SHAP) analysis was conducted to enhance model interpretability, and a web-based tool was subsequently developed based on the best-performing model.

**Results:**

Five key predictive variables—age, sex, body mass index (BMI), uric acid (UA), and alkaline phosphatase (ALP)—were ultimately selected. Among the five models evaluated, the Random Forest model achieved the highest AUC (0.759) in the test set, demonstrating moderate discriminative performance and good model stability. SHAP analysis revealed that BMI contributed most to the model’s predictions, while increased age, female sex, elevated ALP, and reduced UA were associated with a higher risk of osteoporosis. Based on this model, a web-based tool was developed to enable individualized risk prediction and feature-level visualization, providing a quantitative reference for clinical risk assessment.

**Conclusion:**

The osteoporosis prediction model developed in this study achieved quantitative risk estimation and interpretable outputs using a limited set of features, providing a feasible technical approach for early screening of osteoporosis. Future work should focus on external validation and recalibration in multicenter populations to further evaluate and optimize the model’s predictive performance and clinical applicability.

## 1 Introduction

Osteoporosis (OP) is a systemic skeletal disorder characterized by a reduction in bone mineral density (BMD) and the disruption of bone microarchitecture, which together compromise bone strength and increase the risk of fractures. It is considered a major threat to both the quality of life and survival among the elderly population (1). According to the International Osteoporosis Foundation (IOF), approximately one-third of women and one-fifth of men over the age of 50 worldwide are expected to experience at least one osteoporotic fracture during their lifetime, thereby rendering OP a significant global public health concern (2, 3). Given the accelerating pace of population aging, the burden of osteoporosis continues to grow, underscoring the urgent need for early, accurate, and interpretable predictive models to identify high-risk individuals and facilitate timely intervention (4).

Currently, the clinical diagnosis of OP is primarily based on dual-energy X-ray absorptiometry (DXA) to assess BMD, with a T-score ≤ − 2.5 serving as the diagnostic threshold (5). However, the availability of DXA in primary healthcare settings remains limited due to its high cost and technical requirements. Moreover, DXA does not capture the dynamic nature of bone metabolism, limiting its effectiveness in large-scale screening programs (6). Traditional tools, such as the FRAX model, incorporate only a narrow set of clinical risk factors (e.g., age, sex, weight, fracture history), thereby limiting their predictive performance across diverse populations and reflecting a restricted ability to capture the multidimensional regulation of bone metabolism (7, 8). Recent studies have increasingly highlighted that the pathogenesis of OP involves complex interactions among age, sex, endocrine function, inflammatory status, nutritional factors, and numerous biochemical indicators related to bone turnover (9). Serum biochemical and inflammatory markers such as alkaline phosphatase (ALP), uric acid (UA), lymphocyte ratio, serum amyloid A (SAA), and systemic immune-inflammation index (SII) have been shown to be significantly associated with OP risk, providing a theoretical basis for the construction of high-dimensional, multiparameter predictive models (10–12). Nonetheless, identifying the most informative predictors from extensive clinical datasets and developing robust, interpretable models remain core challenges in OP risk modeling.

Machine learning (ML) algorithms have demonstrated substantial potential in medical prediction tasks due to their capacity to handle nonlinear associations, high-dimensional features, and intricate interactions among variables (13). Studies have reported that ML-based models outperform conventional statistical approaches in OP prediction (4, 14). For instance, ML models trained on the NHANES database using algorithms such as XGBoost, LightGBM, and CatBoost identified age, sex, body mass index (BMI), and ALP as key predictors and demonstrated robust generalizability across various validation folds (15). In another study, deep neural networks combined with the LIME explanation algorithm were applied to the Korean KNHANES dataset, achieving high-accuracy prediction of femoral neck BMD (AUC = 0.922) (16). Regarding model interpretability, SHapley Additive exPlanations (SHAP) has gained increasing attention in medical AI research for its ability to generate both global and individual-level attributions, thereby enhancing transparency and trust in model outputs (17, 18). A comparative analysis conducted by Elias et al. found that SHAP outperformed LIME and permutation importance in terms of consistency in feature ranking, local explanation accuracy, and overall model transparency, further reinforcing its clinical applicability (13). Despite these advancements, several challenges persist. Most existing models have been developed using publicly accessible datasets, often without validation in real-world clinical populations, which limits their generalizability. Moreover, many studies rely on univariate statistical tests or expert-driven methods for feature selection, lacking comprehensive variable screening strategies. Furthermore, the absence of user-friendly visualization tools has impeded the clinical translation of such predictive models.

To address these gaps, the present study utilized a decade of real-world clinical data (2015–2024) from the Affiliated Hospital of Jiangxi University of Chinese Medicine, including 5,328 participants. A comprehensive dataset encompassing demographic characteristics, anthropometric measurements, lumbar spine BMD (L1–L4), and over 90 blood biochemical and inflammatory markers was compiled. Through univariate analysis followed by least absolute shrinkage and selection operator (LASSO) regression, five key predictors—age, sex, BMI, UA, and ALP—were identified. Five ML algorithms (Decision Tree, Random Forest, XGBoost, CatBoost, and Multi-Layer Perceptron) were developed and systematically evaluated, with Random Forest selected as the best-performing model. To enhance model interpretability, SHAP was incorporated to provide global and individualized explanations of model predictions. Additionally, a web-based visualization tool was designed, enabling real-time OP risk assessment and SHAP-based interpretation using the five selected input features. This platform offers an intuitive and practical decision support tool for clinicians.

## 2 Methods

### 2.1 Data collection and preprocessing

Clinical data were retrospectively collected from patients treated at the Affiliated Hospital of Jiangxi University of Chinese Medicine over a 10-year period (2015–2024). The dataset was composed of demographic characteristics, anthropometric measurements, lumbar spine bone mineral density (BMD; L1–L4), and a broad spectrum of serum biochemical and inflammatory indicators. Data were extracted from the hospital’s Information Department and the Bone Densitometry Unit. During the data cleaning stage, at the patient level, cases with more than 30% missing clinical variables or missing key variables (such as sex, age, or major laboratory indicators) were excluded from further analysis. At the variable level, clinical indicators with a missing rate exceeding 20% across the total sample were removed if they were non-essential variables. Finally, missing values were imputed using the Random Forest imputation method. Based on the diagnostic criteria for osteoporosis, 5,328 participants were enrolled and subsequently categorized into a non-osteoporotic group (*n* = 3,431) and an osteoporotic group (*n* = 1,897) according to a T-score ≤ − 2.5, in line with World Health Organization (WHO) criteria and the instrument manufacturer’s reference standards (19). Demographic variables comprised age and sex, while anthropometric variables included body mass index (BMI). Biochemical indicators consisted of total protein (TP), albumin (ALB), globulin (GLOB), albumin/globulin (A/G) ratio, cholesterol (CHOL), high-density lipoprotein cholesterol (HDL-C), low-density lipoprotein cholesterol (LDL-C), triglycerides (TG), lipoprotein(a) [LPa], apolipoprotein B (APO-B), apolipoprotein A1 (APO-A1), total bilirubin (TBIL), direct bilirubin (DBIL), indirect bilirubin (IBIL), total bile acid (TBA), alkaline phosphatase (ALP), aspartate aminotransferase (AST), alanine aminotransferase (ALT), AST/ALT ratio, *γ*-glutamyl transferase (GGT), alpha-L-fucosidase (AFU), white blood cell count (WBC), absolute and percentage values of neutrophils (NEUT#/%), lymphocytes (LYMPH#/%), monocytes (MONO#/%), eosinophils (EOS#/%), and basophils (BASO#/%). Additional variables included red blood cell count (RBC), hemoglobin (HGB), hematocrit (HCT), mean corpuscular volume (MCV), mean corpuscular hemoglobin (MCH), mean corpuscular hemoglobin concentration (MCHC), red cell distribution width-standard deviation (RDW-SD), red cell distribution width-coefficient of variation (RDW-CV), platelet count (PLT), plateletcrit (PCT), platelet distribution width (PDW), mean platelet volume (MPV), platelet-large cell ratio (P-LCR), glucose (GLU), uric acid (UA), urea (UREA), sodium (Na), chloride (Cl), potassium (K), phosphorus (P), calcium (Ca), complement C1q, serum amyloid A (SAA), serum sialic acid (SA), β2-microglobulin (β2-MG), and adenosine deaminase (ADA). In addition, derived ratios and composite indices included neutrophil-to-lymphocyte ratio (NLR), platelet-to-lymphocyte ratio (PLR), lymphocyte-to-monocyte ratio (LMR), neutrophil-to-platelet ratio (NPR), platelet-to-albumin ratio (PAR), systemic inflammatory response index (SIRI), systemic immune-inflammation index (SII), uric acid to HDL-C ratio (UHR), cholesterol-to-HDL-C ratio (TC/HDL-C), and hemoglobin-to-RDW ratio (HRR). This study was a single-center retrospective analysis, in which model development and internal validation were conducted using data from the same source. The absence of external independent cohort validation may limit the model’s generalizability to other regions and diverse healthcare populations. The study protocol was approved by the Ethics Committee of the Affiliated Hospital of Jiangxi University of Chinese Medicine (Approval No. JZFYLL20220727032).

### 2.2 Feature selection using LASSO regression

To identify key features associated with OP, least absolute shrinkage and selection operator (LASSO) regression was applied to variables demonstrating statistical significance (*p* < 0.05) in the baseline analysis. The optimal penalty parameter (*λ*) was determined using ten-fold cross-validation. Features with non-zero coefficients were retained for subsequent model construction.

### 2.3 Machine learning model construction and validation

Five machine learning (ML) models—Decision Tree, Random Forest, XGBoost, CatBoost, and Multi-Layer Perceptron (MLP)—were constructed and comparatively evaluated. The dataset was randomly partitioned into a training set (70%) and a test set (30%). Models were trained using the training set and evaluated on the test set. Model performance was assessed using area under the receiver operating characteristic curve (AUC), accuracy, precision, recall, F1-score, positive predictive value (PPV), and negative predictive value (NPV). The model demonstrating the highest AUC and strongest generalizability was selected as the final predictive model and subsequently subjected to 10-fold cross-validation across the training, validation, and test sets to evaluate its robustness and predictive performance. To evaluate the clinical applicability of the model, a decision curve analysis (DCA) was further conducted to compare the optimal machine learning model with the optimal model built on a simple clinical rule comprising age, sex, and BMI. By comparing the two DCA curves, the net clinical benefit of the machine learning model over the traditional clinical rule model was quantitatively assessed.

### 2.4 Model interpretation using SHAP and LIME

To improve interpretability, SHapley Additive exPlanations (SHAP) was utilized to generate both global and local explanations for the optimal predictive model. Mean SHAP values were computed to quantify the contribution of each feature to the model output. In addition, SHAP force plots were generated for representative samples to visualize individual-level feature importance and the directionality of their effects. To further validate the local interpretability results, the Local Interpretable Model-agnostic Explanations (LIME) algorithm was additionally applied. LIME constructs locally linear surrogate models around individual predictions by perturbing the input data and observing corresponding output changes, thereby estimating each feature’s local contribution to the prediction. For representative cases, LIME explanation maps were visualized to compare the probability contributions of each variable toward osteoporosis and non-osteoporosis classifications, providing complementary interpretive evidence to the SHAP analysis.

### 2.5 Web-based visualization tool development

An interactive web-based tool was developed using the optimal model to facilitate individualized OP risk prediction. By inputting individual feature values, real-time predictions of OP probability are generated along with SHAP-based visual explanations. This functionality thereby offers clinicians a transparent and intuitive decision-support interface.

### 2.6 Statistical analysis

Statistical analyses were performed using R software (v4.2.1) and Python (v3.9). Normally distributed variables were reported as mean ± standard deviation (SD) and compared using the independent samples *t*-test. Non-normally distributed variables were presented as median (interquartile range, IQR) and analyzed using the Mann–Whitney U test. Categorical variables were expressed as frequency (percentage) and compared using the chi-square (χ^2^) test. Variables with *p* < 0.05 were included in least absolute shrinkage and selection operator (LASSO) regression for feature selection. Model performance was assessed using area under the receiver operating characteristic curve (AUC), accuracy, precision, recall, and F1-score. Ten-fold cross-validation was conducted to assess model stability. SHapley Additive exPlanations (SHAP) was applied for model interpretability analysis. A two-tailed *p*-value < 0.05 was considered statistically significant.

## 3 Results

### 3.1 Baseline characteristics

**Figure 1:**
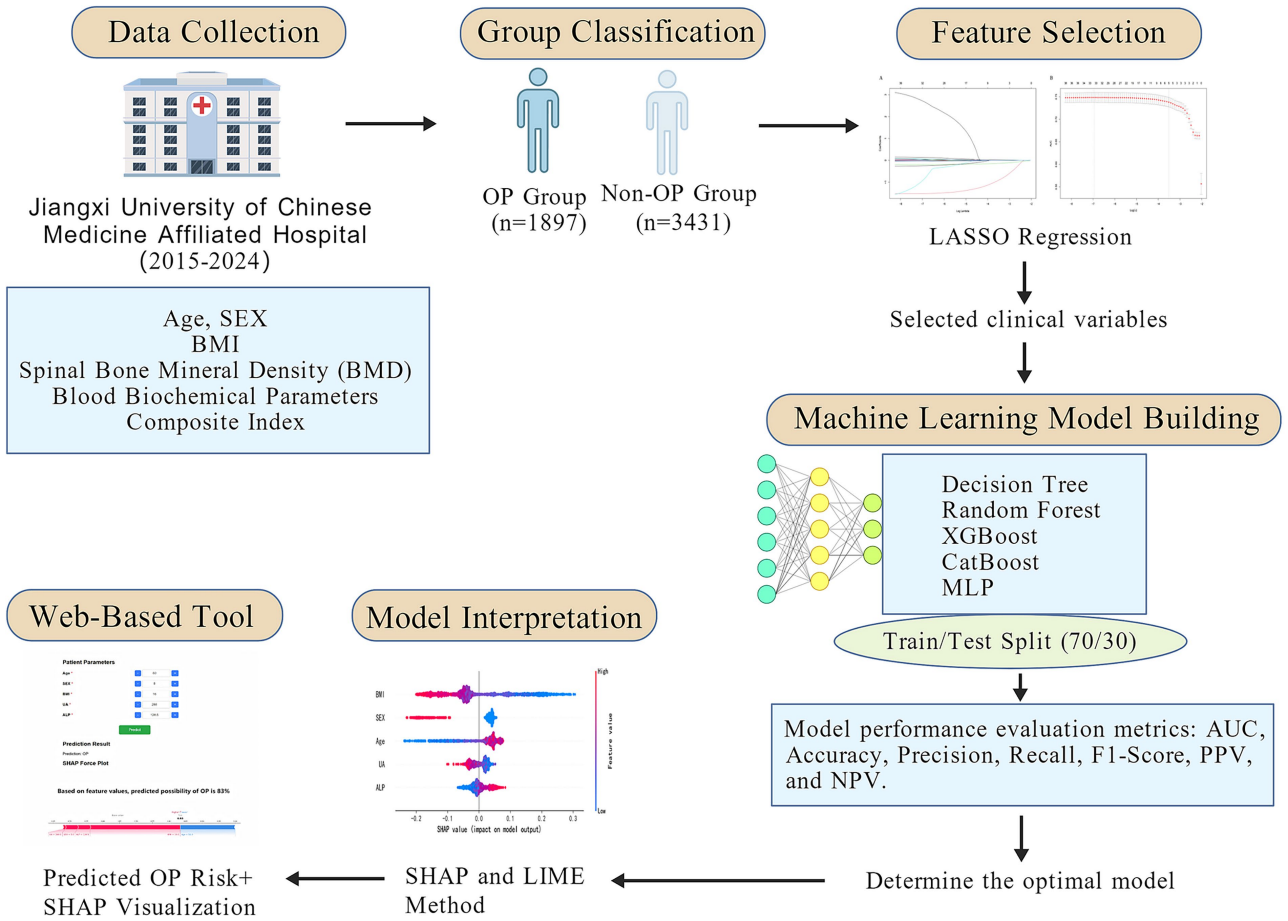
Workflow diagram of the study.

The overall study workflow is illustrated in Figure 1. A total of 5,328 patients were enrolled and classified into the non-osteoporosis (non-OP) group (*n* = 3,431) and the osteoporosis (OP) group (*n* = 1,897). Of the total cohort, 939 were male (17.6%) and 4,389 were female (82.4%). Compared with the non-OP group, patients in the OP group were significantly older (*p* < 0.001), had a higher proportion of females (*p* < 0.001), and exhibited lower BMI (*p* < 0.001). Laboratory findings revealed significantly elevated levels of NEUT%, LPa, SA, SAA, and ADA in the OP group (all *p* < 0.001), whereas PDW was markedly decreased (*p* < 0.001). Furthermore, significantly lower values were observed in HGB, EOS#, EOS%, LYMPH#, LYMPH%, HCT, RBC, ALT, GGT, TG, UA, P-LCR, WBC, ALB, and AFU in the OP group (all *p* < 0.001). Levels of AST/ALT, ALP, HDL-C, MONO%, NLR, PLR, PAR, SIRI, and SII were significantly elevated (all *p* < 0.001), while HRR, LMR, UHR, and TC/HDL-C were significantly decreased in the OP group (all *p* < 0.001). Additional significant differences were observed in TP, potassium (K), MCHC, A/G ratio, and BASO# between the two groups (*p* < 0.05). A total of 42 clinical variables with *p* < 0.05 were identified through baseline comparisons and selected for further analysis (Table 1; Figure 1).

**Table 1:** Baseline characteristics of study participants.

Variable	Non-OP group (*N* = 3,431)	OP group (*N* = 1897)	*P*
Age (years)	68.0 [59.0; 76.0]	71.0 [65.0; 78.0]	<0.001
Sex			<0.001
Female	2,641 (77.0%)	1748 (92.1%)	
Male	790 (23.0%)	149 (7.85%)	
BMI (kg/m^2^)	23.8 [21.7; 26.2]	21.9 [19.6; 24.2]	<0.001
TP (g/L)	68.9 [63.8; 73.2]	68.1 [63.2; 72.8]	0.015
TBA (umol/L)	5.90 [3.00; 10.0]	5.80 [3.30; 9.80]	0.393
TBIL (umol/L)	11.2 [8.64; 14.9]	11.2 [8.66; 15.0]	0.562
CHOL (mmol/L)	4.74 [4.00; 5.47]	4.69 [4.00; 5.47]	0.847
NEUT# (10^9/L)	3.85 [2.92; 4.76]	3.81 [2.81; 4.86]	0.619
NEUT% (%)	64.9 [57.2; 70.2]	66.7 [58.1; 72.4]	<0.001
DBIL (umol/L)	4.20 [3.07; 5.76]	4.30 [3.14; 5.94]	0.102
LPa (mg/dL)	110 [46.0; 231]	125 [51.0; 258]	<0.001
APO-B (g/L)	0.94 [0.72; 1.16]	0.92 [0.72; 1.13]	0.194
APO-A1 (g/L)	1.42 [1.18; 1.72]	1.44 [1.20; 1.73]	0.132
PCT (%)	0.22 [0.18; 0.25]	0.22 [0.18; 0.25]	0.5
PDW (%)	15.9 [15.1; 16.3]	15.8 [14.5; 16.2]	<0.001
PLT (10^9/L)	212 [175; 244]	213 [174; 249]	0.295
SA (mg/dl)	56.6 [52.2; 63.6]	57.5 [52.2; 65.0]	<0.001
SAA (mg/L)	6.60 [3.28; 22.5]	8.80 [3.97; 46.3]	<0.001
HGB (g/L)	125 [116; 133]	122 [112; 129]	<0.001
β2-M2 (mg/L)	2.20 [1.81; 2.61]	2.20 [1.80; 2.70]	0.527
ADA (U/L)	9.70 [6.70; 12.8]	10.3 [7.20; 13.3]	<0.001
EOS# (10^9/L)	0.10 [0.06; 0.15]	0.09 [0.05; 0.13]	<0.001
EOS% (%)	1.66 [1.00; 2.60]	1.50 [0.80; 2.50]	<0.001
BASO# (10^9/L)	0.02 [0.02; 0.03]	0.02 [0.01; 0.03]	0.022
BASO% (%)	0.30 [0.20; 0.50]	0.30 [0.20; 0.50]	0.249
GLOB (g/L)	26.3 [23.3; 29.7]	26.3 [23.4; 29.8]	0.464
GLU (mmol/L)	5.37 [4.87; 6.36]	5.30 [4.79; 6.20]	0.002
MPV (fL)	10.1 [9.60; 11.0]	10.1 [9.60; 11.0]	0.591
MCHC (g/L)	333 [327; 338]	332 [327; 338]	0.003
MCH (pg)	30.6 [29.6; 31.6]	30.5 [29.6; 31.6]	0.684
UA (umol/L)	297 [245; 358]	272 [226; 328]	<0.001
UREA (mmol/L)	5.54 [4.53; 6.70]	5.45 [4.43; 6.90]	0.583
NA (mmol/L)	140 [138; 142]	140 [138; 142]	0.546
CL (mmol/L)	103 [100; 106]	103 [100; 106]	0.735
P (mmol/L)	1.17 [1.05; 1.28]	1.16 [1.05; 1.29]	0.578
LYMPH# (10^9/L)	1.48 [1.19; 1.88]	1.42 [1.07; 1.73]	<0.001
LYMPH% (%)	25.4 [20.6; 32.8]	23.5 [18.3; 32.2]	<0.001
ALP (U/L)	77.7 [63.8; 95.9]	83.0 [68.0; 104]	<0.001
IBIL (umol/L)	6.90 [5.18; 9.50]	6.95 [5.03; 9.30]	0.66
K (mmol/L)	4.10 [3.80; 4.30]	4.00 [3.80; 4.30]	0.004
HCT (%)	37.6 [34.9; 39.8]	36.7 [34.0; 38.8]	<0.001
MCV (fL)	91.7 [89.4; 94.2]	91.9 [89.4; 94.5]	0.177
RDW-SD (fL)	43.2 [41.7; 45.1]	43.2 [41.7; 45.1]	0.776
RDW-CV (%)	12.9 [12.5; 13.4]	12.9 [12.5; 13.4]	0.086
RBC (10^12/L)	4.12 [3.80; 4.41]	4.02 [3.67; 4.31]	<0.001
AST (U/L)	19.4 [16.0; 23.9]	19.3 [16.1; 23.4]	0.7
AST/ALT	1.25 [0.99; 1.55]	1.39 [1.08; 1.73]	<0.001
ALT (U/L)	15.0 [11.2; 21.3]	13.5 [10.1; 18.9]	<0.001
GGT (U/L)	21.6 [16.0; 32.2]	20.0 [14.8; 28.3]	<0.001
HDL-C (mmol/L)	1.36 [1.12; 1.67]	1.43 [1.18; 1.75]	<0.001
TG (mmol/L)	1.24 [0.90; 1.80]	1.12 [0.83; 1.55]	<0.001
CA (mmol/L)	2.30 [2.18; 2.40]	2.29 [2.18; 2.39]	0.107
LDL-c (mmol/l)	2.90 [2.23; 3.56]	2.84 [2.19; 3.50]	0.165
MONO# (10^9/L)	0.39 [0.30; 0.47]	0.39 [0.30; 0.48]	0.663
MONO% (%)	6.50 [5.40; 7.54]	6.60 [5.50; 8.00]	<0.001
P-LCR (%)	26.5 [22.6; 33.1]	26.4 [22.0; 32.6]	0.273
C1q (mg/L)	195 [175; 212]	196 [175; 215]	0.124
WBC (10^9/L)	6.07 [4.91; 7.13]	5.85 [4.73; 7.00]	0.001
A/G	1.60 [1.40; 1.80]	1.60 [1.40; 1.80]	0.008
ALB (g/L)	42.1 [38.9; 44.8]	41.4 [38.4; 44.3]	<0.001
AFU (U/L)	20.2 [16.4; 24.0]	19.6 [16.0; 23.5]	<0.001
NLR	2.54 [1.75; 3.38]	2.81 [1.82; 3.99]	<0.001
PLR	142 [108; 175]	149 [115; 197]	<0.001
LMR	3.84 [2.98; 5.40]	3.51 [2.62; 5.05]	<0.001
NPR	0.02 [0.01; 0.02]	0.02 [0.01; 0.02]	0.413
PAR	4.99 [4.13; 5.88]	5.07 [4.23; 6.07]	0.01
SIRI	0.98 [0.57; 1.49]	1.10 [0.61; 1.65]	<0.001
SII	539 [341; 727]	594 [357; 844]	<0.001
UHR	218 [162; 296]	189 [146; 253]	<0.001
TC.HDL.C	3.45 [2.85; 4.13]	3.26 [2.69; 3.90]	<0.001
HRR	9.69 [8.77; 10.4]	9.47 [8.56; 10.2]	<0.001

### 3.2 Feature selection using LASSO regression

**Figure 2:**
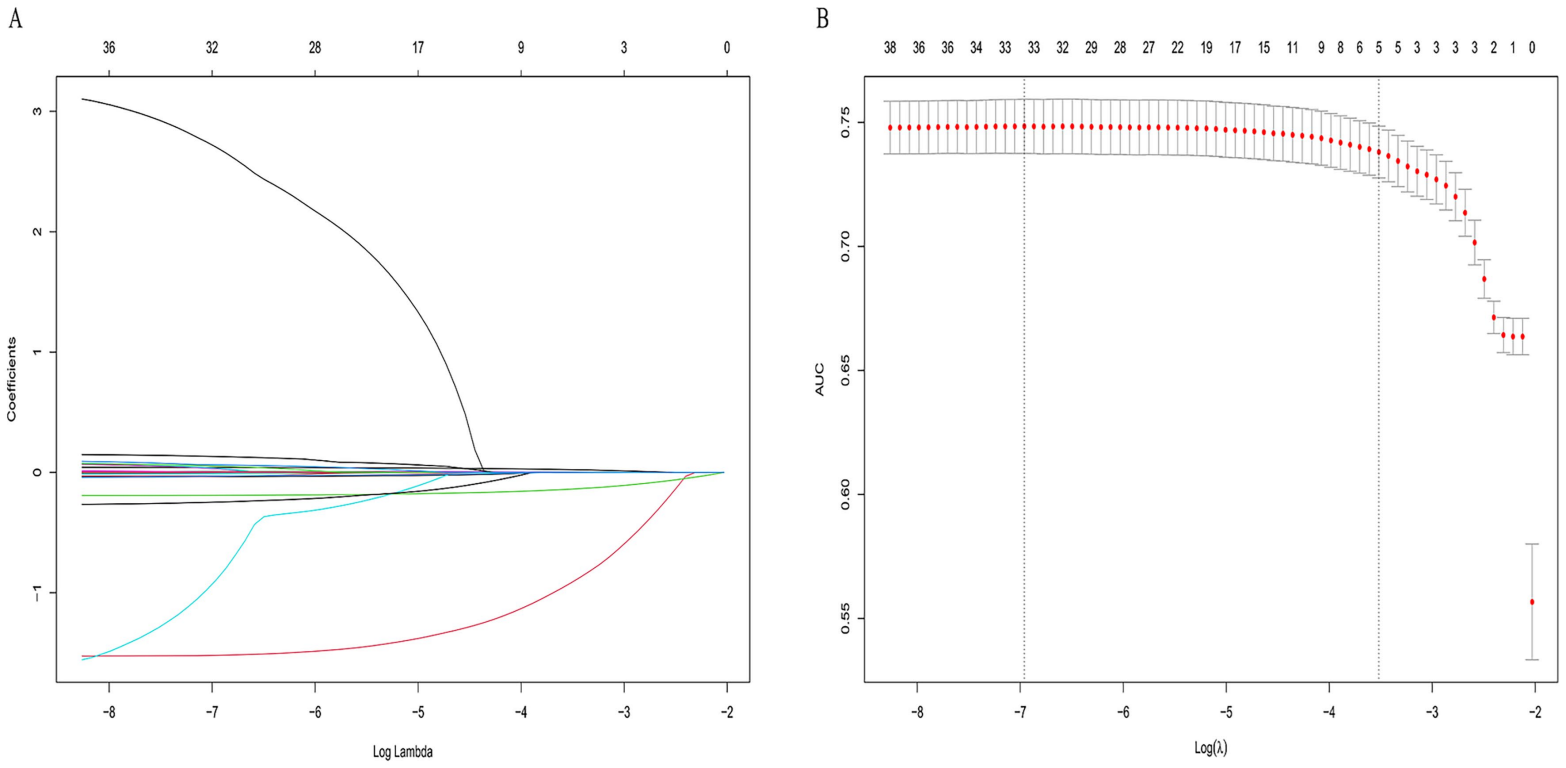
LASSO regression for feature selection in osteoporosis prediction. **(A)** Path of coefficients as a function of the regularization parameter *λ*. **(B)** Ten-fold cross-validation for optimal λ selection. Five non-zero coefficient variables—age, sex, MI, UA, and ALP—were selected.

Clinical variables included in the analysis encompassed demographic data, anthropometric measures, and a broad range of biochemical parameters. Based on the 42 significant features identified from baseline analysis, LASSO regression was employed to select the most predictive features. Five variables with non-zero coefficients were ultimately retained: age, sex, BMI, UA, and ALP (Figure 2). The optimal *λ* value of the LASSO regression was 0.0296. In the ten-fold cross-validation, Age, Sex, BMI, UA, and ALP consistently retained non-zero coefficients across all folds, with a selection frequency of 100%, indicating high feature selection consistency and model stability.

### 3.3 Construction and selection of machine learning models

**Figure 3:**
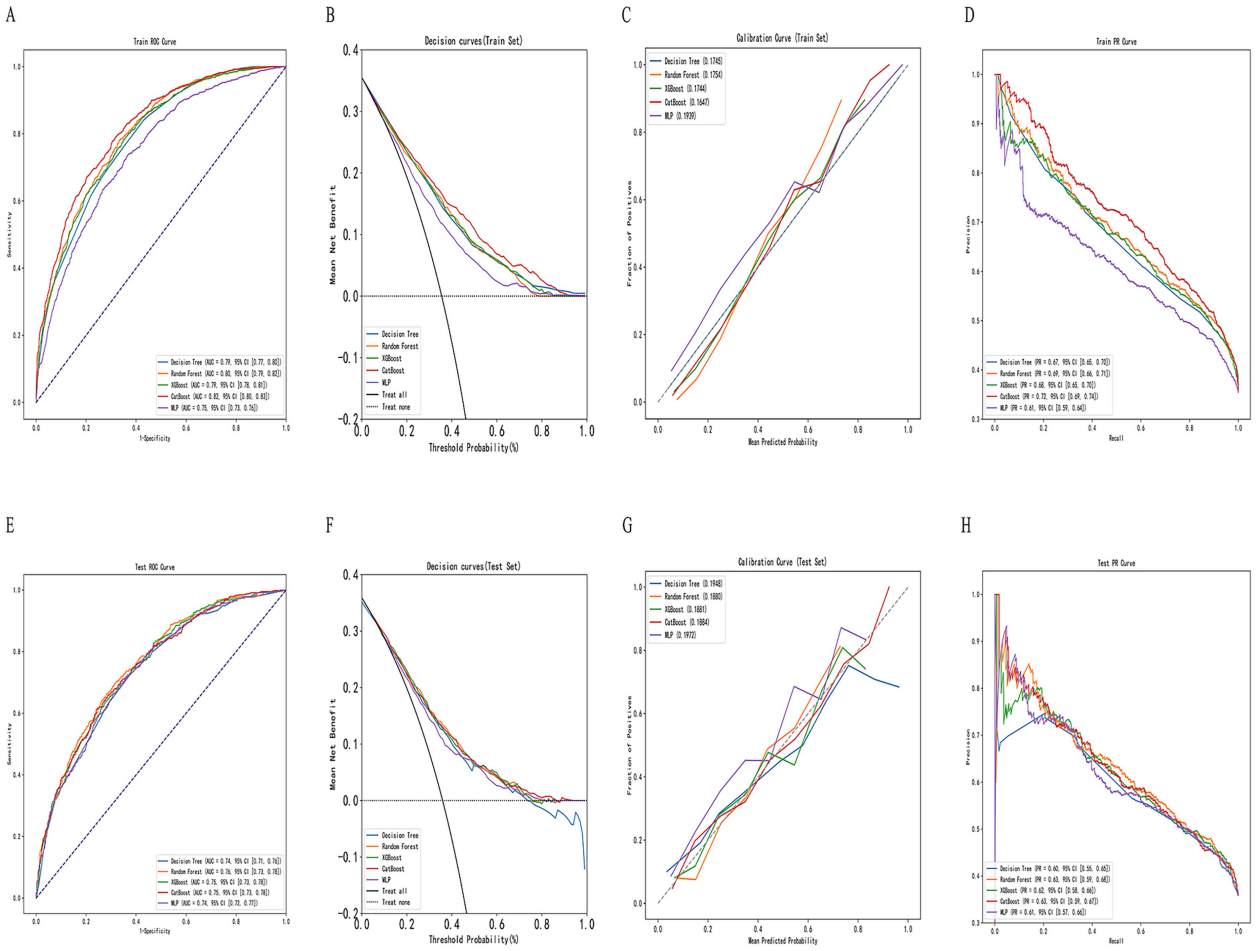
Construction and evaluation of machine learning models. **(A,E)** Receiver operating characteristic (ROC) curves for training and testing sets, respectively. **(B,F)** Decision curve analysis (DCA) for net clinical benefit evaluation. **(C,G)** Calibration curves for assessing model agreement between predicted and actual probabilities. **(D,H)** Precision–recall (PR) curves for evaluating classification performance across thresholds.

**Figure 4:**
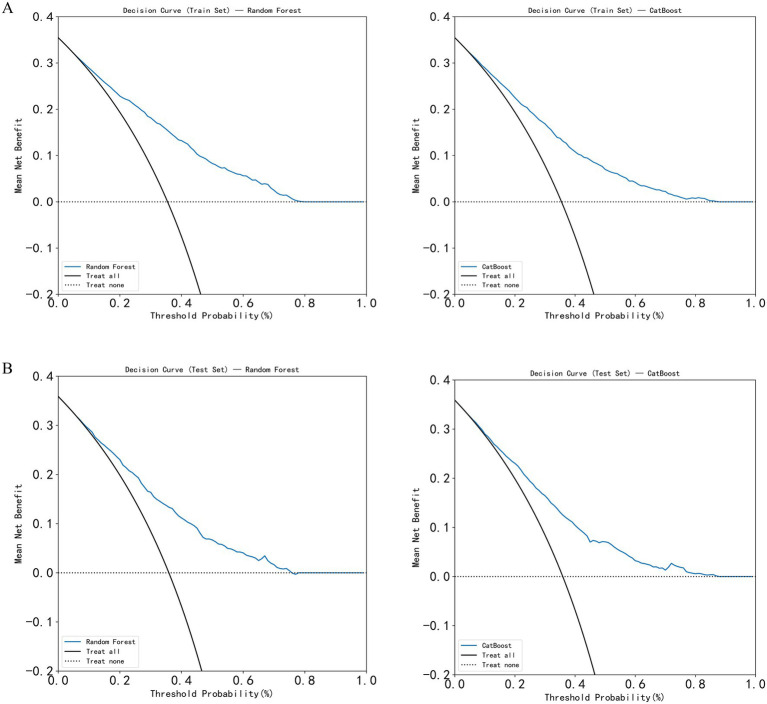
Decision curve analysis (DCA) of the models. **(A)** DCA curves of the Random Forest and CatBoost models in the training set; **(B)** DCA curves of the Random Forest and CatBoost models in the test set.

In this study, five machine learning models—Decision Tree, Random Forest, XGBoost, CatBoost, and MLP—were constructed and compared for predictive performance in both the training and test sets. In the training set, the CatBoost model demonstrated superior performance, achieving an AUC of 0.818 (95% CI: 0.710–0.824) and an accuracy of 0.754 (95% CI: 0.709–0.825), followed by the Random Forest model with an AUC of 0.802 (95% CI: 0.694–0.809). In the test set, the Random Forest model achieved the highest AUC (0.759, 95% CI: 0.645–0.770), followed by XGBoost (0.753, 95% CI: 0.624–0.765) and CatBoost (0.752, 95% CI: 0.616–0.764). Considering both training and test results, the Random Forest model exhibited stable performance across datasets without evident overfitting or performance fluctuation. Given its consistent and moderate predictive capability in the test set, the Random Forest model was selected as the primary model for subsequent analyses (Table 2; Figure 3). In the simple clinical rule model constructed with age, sex, and BMI, the CatBoost algorithm achieved the best performance in the test set, with an AUC of 0.747 (95% CI: 0.650–0.758), and was identified as the optimal model for the Age + Sex + BMI group. The DCA results indicated that both the Random Forest and CatBoost models provided higher net benefits than the treat-all and treat-none strategies in both the training and test sets. Compared with the optimal simple clinical rule model (CatBoost), the optimal machine learning model (Random Forest) demonstrated greater net benefit and a wider range of effective threshold probabilities across most thresholds, suggesting superior clinical applicability for individualized osteoporosis risk assessment (Figure 4).

**Table 2:** Performance comparison of machine learning classifiers for OP risk prediction.

Group	Model	AUC (95% CI)	Accuracy (95% CI)	Precision (95% CI)	Recall (95% CI)	F1-score (95% CI)	PPV (95% CI)	NPV (95% CI)
Train group	Decision Tree	0.788 (0.685, 0.795)	0.726 (0.686, 0.795)	0.729 (0.685, 0.795)	0.726 (0.685, 0.794)	0.694 (0.685, 0.795)	0.741 (0.686, 0.794)	0.723 (0.686, 0.795)
	Random Forest	0.802 (0.694, 0.809)	0.729 (0.694, 0.808)	0.728 (0.694, 0.808)	0.729 (0.693, 0.808)	0.703 (0.694, 0.809)	0.725 (0.694, 0.809)	0.73 (0.694, 0.808)
	XGBoost	0.794 (0.693, 0.801)	0.732 (0.691, 0.801)	0.727 (0.693, 0.801)	0.732 (0.692, 0.801)	0.711 (0.692, 0.801)	0.707 (0.692, 0.801)	0.738 (0.692, 0.801)
	CatBoost	0.818 (0.710, 0.824)	0.754 (0.709, 0.825)	0.75 (0.708, 0.824)	0.754 (0.709, 0.825)	0.741 (0.708, 0.825)	0.723 (0.710, 0.824)	0.765 (0.710, 0.825)
	MLP	0.749 (0.653, 0.757)	0.703 (0.654, 0.757)	0.699 (0.654, 0.756)	0.703 (0.654, 0.756)	0.664 (0.654, 0.757)	0.688 (0.653, 0.757)	0.705 (0.653, 0.757)
Test group	Decision Tree	0.739 (0.656, 0.751)	0.707 (0.653, 0.750)	0.704 (0.654, 0.751)	0.707 (0.655, 0.751)	0.675 (0.655, 0.751)	0.691 (0.657, 0.750)	0.711 (0.654, 0.751)
	Random Forest	0.759 (0.645, 0.770)	0.707 (0.646, 0.770)	0.7 (0.646, 0.769)	0.707 (0.645, 0.771)	0.68 (0.647, 0.770)	0.671 (0.647, 0.770)	0.716 (0.647, 0.770)
	XGBoost	0.753 (0.624, 0.765)	0.702 (0.623, 0.764)	0.692 (0.624, 0.764)	0.702 (0.625, 0.765)	0.678 (0.623, 0.765)	0.648 (0.620, 0.763)	0.717 (0.625, 0.764)
	CatBoost	0.752 (0.616, 0.764)	0.71 (0.617, 0.765)	0.7 (0.616, 0.764)	0.71 (0.617, 0.764)	0.696 (0.615, 0.764)	0.639 (0.617, 0.764)	0.734 (0.619, 0.764)
	MLP	0.743 (0.653, 0.758)	0.706 (0.654, 0.757)	0.707 (0.654, 0.757)	0.706 (0.655, 0.758)	0.668 (0.654, 0.758)	0.71 (0.654, 0.757)	0.705 (0.654, 0.758)

### 3.4 Ten-fold cross-validation of the optimal machine learning model

**Figure 5:**
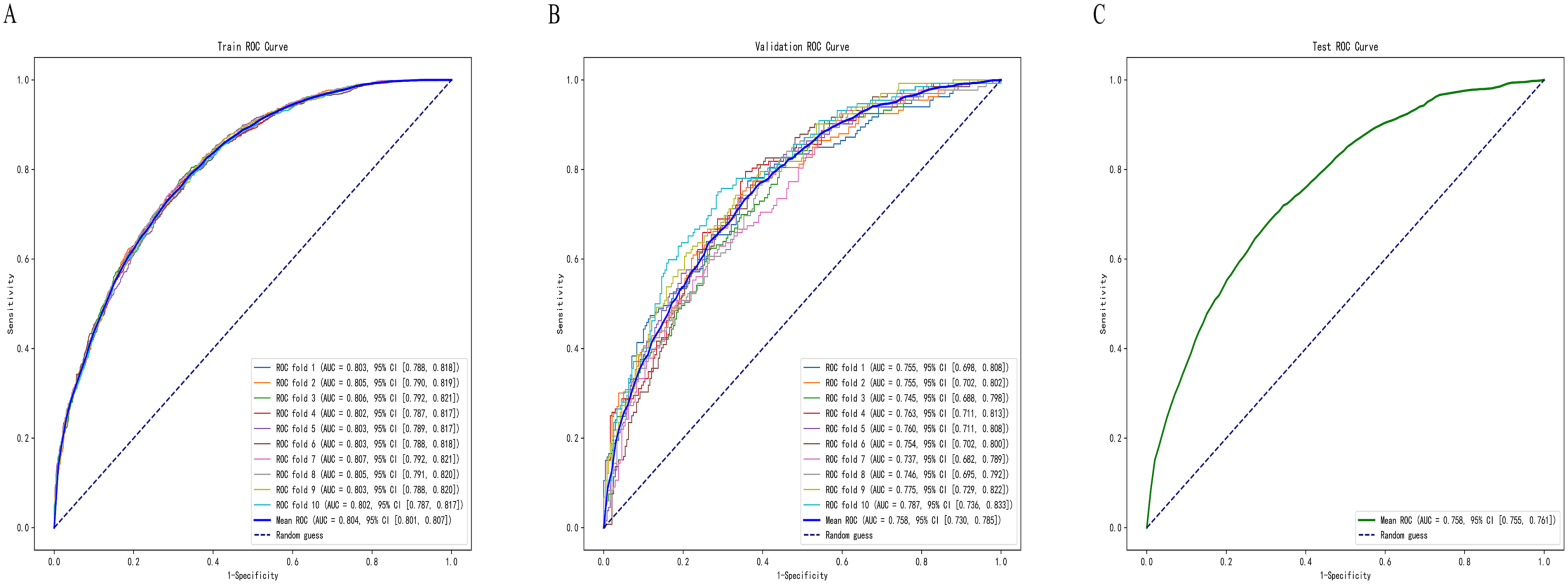
ROC curves of the Random Forest model on the **(A)** training set, **(B)** validation set, and **(C)** test set.

Random Forest was identified as the optimal machine learning (ML) model. To further assess its predictive accuracy and robustness, ten-fold cross-validation was conducted. The entire dataset was randomly split in a stratified manner, with 70% of the samples assigned to the training set and 30% to the independent test set, while maintaining the same proportion of positive (osteoporosis) and negative (non-osteoporosis) cases in both subsets. Ten-fold cross-validation was then performed within the training data to optimize hyperparameters and evaluate internal performance. The Random Forest model achieved an area under the receiver operating characteristic curve (AUC) of 0.804 (95% CI: 0.801–0.807) on the training set, 0.758 (95% CI: 0.730–0.785) on the validation set, and 0.758 (95% CI: 0.755–0.761) on the test set (Figure 5). The results indicated that the model demonstrated stable performance during internal validation and exhibited a certain degree of generalizability. Its predictive performance was at a moderate level, warranting further validation using external datasets.

### 3.5 Model interpretability based on SHAP and LIME analyses

**Figure 6:**
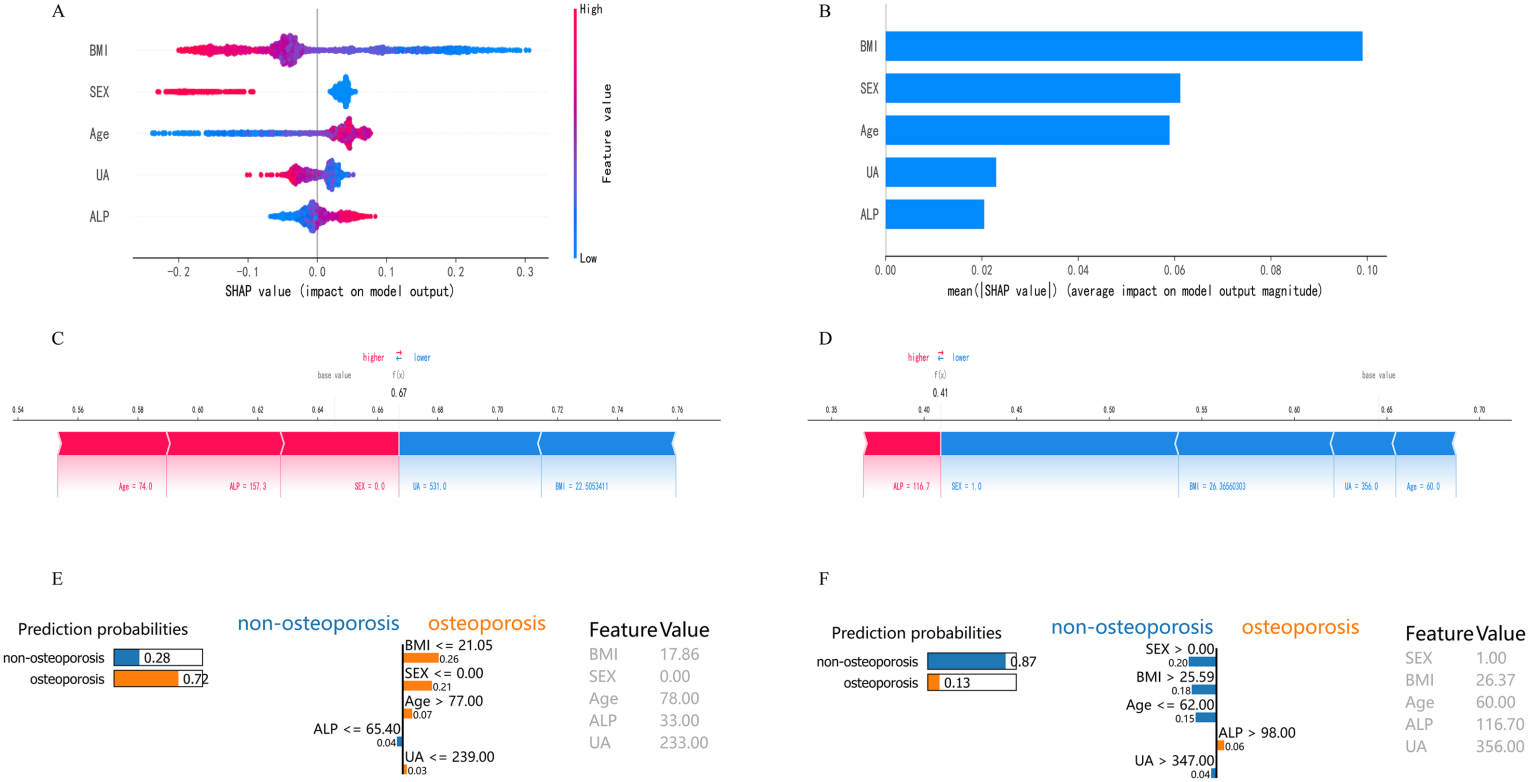
Model interpretability based on SHAP and LIME analyses. **(A)** SHAP summary plot of feature contributions. **(B)** Bar plot of mean absolute SHAP values. **(C)** SHAP force plot for a typical high-risk patient (f(x) = 0.67). **(D)** SHAP force plot for a typical low-risk patient (f(x) = 0.41). **(E)** LIME explanation plot for a representative high-risk individual. The bars show each variable’s local contribution to the predicted probability of osteoporosis. Lower BMI, advanced age, female sex, decreased UA, and elevated ALP jointly increased the probability of osteoporosis (0.72). **(F)** LIME explanation plot for a representative low-risk individual. Higher BMI, male sex, and higher UA contributed to a reduced probability of osteoporosis (0.13).

Figure 6 displays the SHapley Additive exPlanations (SHAP) visualization results for the Random Forest model. SHAP facilitates interpretation of model outputs by quantifying the contribution of each feature to individual predictions. Lower BMI, female sex, older age, decreased uric acid (UA), and elevated alkaline phosphatase (ALP) were associated with increased osteoporosis (OP) risk, thereby identifying them as key risk factors (Figure 6A). Based on mean SHAP values, BMI contributed most to the model’s predictive output, followed by sex, age, UA, and ALP (Figure 6B). SHAP force plots were used to illustrate individual-level predictions for two representative cases (Figures 6C,D). For Patient 1 (f(x) = 0.67), the SHAP force plot showed the following values: age = 74.0, ALP = 157.3, sex = 0 (female), BMI = 22.51, and UA = 531.0. Age and ALP exhibited positive contributions to OP risk prediction, whereas BMI and UA contributed negatively, thereby reducing the predicted risk. For Patient 2 (f(x) = 0.41), the values were ALP = 116.7, sex = 1 (male), BMI = 26.37, UA = 356.0, and age = 60.0. In this case, ALP contributed positively to risk, while BMI, UA, and age contributed negatively. These individual-level SHAP analyses confirmed the relevance and directional impact of the five selected features—BMI, sex, age, UA, and ALP—in predicting OP, thereby supporting the interpretability of the model. LIME analysis provided additional case-level interpretability for the model. As shown in Figures 6E,F, the LIME explanation plots presented the predicted probabilities of osteoporosis and non-osteoporosis for representative individuals, together with the contribution weights of each variable. In high-risk cases (Figure 6E), lower BMI, advanced age, female sex, decreased uric acid, and elevated alkaline phosphatase were the main drivers that increased the predicted probability of osteoporosis (0.72). Conversely, in low-risk cases (Figure 6F), higher BMI, male sex, and higher uric acid contributed to a reduced probability of osteoporosis (0.13). The LIME results were largely consistent with the SHAP interpretations, confirming that BMI, sex, and age exerted the strongest local influence on prediction outcomes, while UA and ALP provided complementary information. This dual-model interpretive framework strengthens the reliability and transparency of the model’s decision process.

### 3.6 Development of a web-based visualization tool

**Figure 7:**
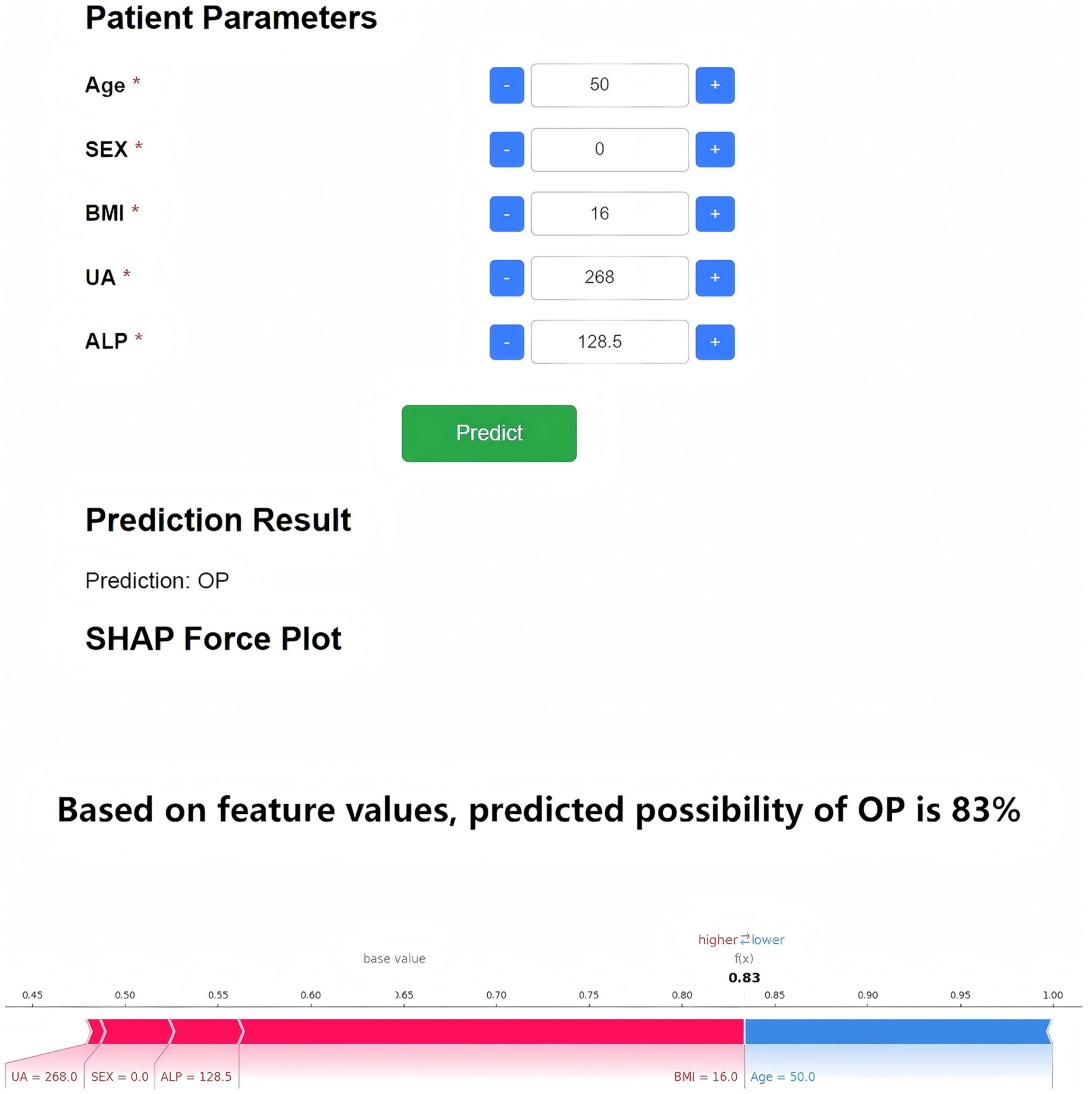
Web-based OP risk prediction and SHAP interpretability tool. The deployed application based on the five-feature Random Forest model enables real-time OP risk prediction. Upon data entry, the tool provides a probability estimate and an individualized SHAP force plot. Blue features shift the model toward non-OP, while red features indicate OP risk factors.

**Figure 8:**
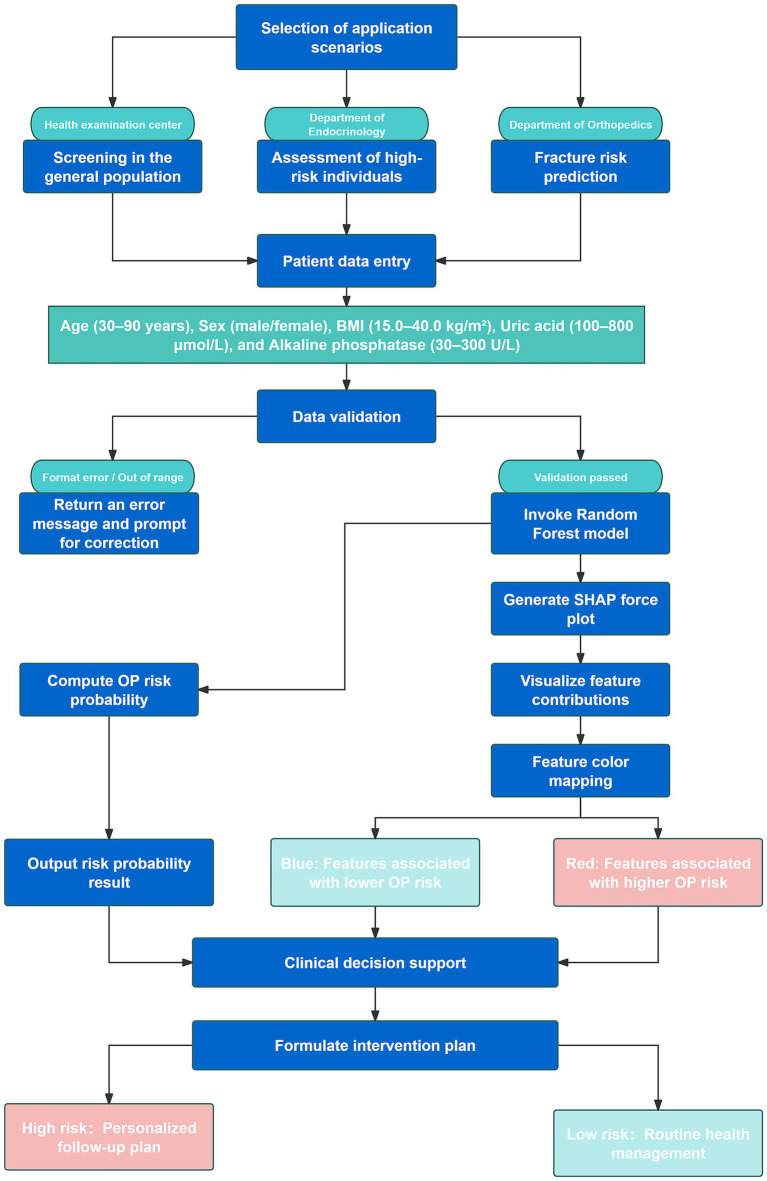
Flowchart illustrating the clinical integration process and application scenarios of the web-based Random Forest osteoporosis risk prediction model.

To enhance clinical applicability, the final Random Forest model was deployed as a web-based application (Figure 7). By inputting the five required feature values, the tool generates an immediate OP risk probability for the individual patient. Additionally, a personalized SHAP force plot is produced, visually illustrating the contribution of each feature to the prediction. In the SHAP visualization, blue-colored features shift the prediction toward the non-OP category, while red-colored features increase the likelihood of OP. For instance, when a patient’s predicted OP probability is 83%, the SHAP plot highlights the primary risk-enhancing and risk-reducing features, thereby improving transparency in clinical decision-making. This study developed an online osteoporosis risk calculator based on the Flask framework and HTML5, which can be directly accessed through a web browser without additional installation. The tool integrates five key variables—age (30–90 years), sex (male/female), BMI (15.0–40.0 kg/m^2^), uric acid (100–800 μmol/L), and alkaline phosphatase (30–300 U/L)—with built-in input range checks, format constraints, and logical validation to ensure data integrity and reliability of results. It is currently undergoing usability evaluation and functional optimization. Although not yet implemented in clinical pilot settings, its modular design enables future integration as an independent module within hospital electronic medical record or health examination management systems. The tool is intended for deployment in health examination centers, endocrinology departments, and orthopedic clinics to provide physicians with real-time, visualized risk scoring and feature interpretation for stratified screening and personalized follow-up planning (Figure 8).

## 4 Discussion

Osteoporosis (OP) is a chronic metabolic bone disorder characterized by decreased bone mineral density (BMD) and deterioration of bone microarchitecture, which significantly increases the risk of fragility fractures, especially in the elderly population (20). Conventional diagnostic methods, such as dual-energy X-ray absorptiometry (DXA), are often constrained by cost and limited accessibility. Additionally, widely used tools such as FRAX lack the capacity to capture complex, multidimensional clinical data (21). The present study aimed to develop an OP risk prediction model using high-dimensional real-world clinical data to improve the accuracy and efficiency of early screening. This study introduces three primary innovations. First, a large real-world dataset from the Affiliated Hospital of Jiangxi University of Chinese Medicine was utilized, comprising over 5,000 patients and integrating multi-level clinical characteristics and biochemical indicators. Second, a combined approach involving least absolute shrinkage and selection operator (LASSO) regression and machine learning algorithms was employed to identify key predictors and construct a robust risk assessment model. Third, SHapley Additive exPlanations (SHAP) were integrated to enhance model interpretability, thereby improving the transparency and clinical applicability of the prediction results. The results showed that the Random Forest model achieved an AUC of 0.759 on the test set, indicating a moderate discriminative ability. Among the selected predictors, body mass index (BMI), sex, age, uric acid (UA), and alkaline phosphatase (ALP) were identified as the most influential features. These variables align with previously reported risk factors for OP and further support their relevance in osteoporosis risk assessment.

A systematic comparison was conducted to evaluate the performance of five machine learning (ML) models for predicting osteoporosis (OP) risk. Considering the combined results from the training and test sets, the Random Forest model demonstrated stable performance across datasets without evident overfitting or performance fluctuation. Given its consistent and moderate predictive capability in the test set, the Random Forest model was selected as the primary model for subsequent analyses. For comparison, Wu et al. reported an XGBoost model with an AUC of 0.890 and an accuracy of 0.902 for OP prediction (22), while Je et al. achieved an AUC of 0.86 and an accuracy of 0.85 using the same algorithm (14). The AUC obtained by the Random Forest model in the present study is comparable to these findings, thereby supporting its clinical validity. Furthermore, Elias et al. observed that XGBoost performs well across multiple evaluation metrics, including accuracy, precision, and F1-score (13). Taken together, although CatBoost exhibited strong training-phase performance, Random Forest demonstrated the most consistent and generalizable results in the test set and was therefore selected as the final predictive model.

LASSO regression was employed in this study to identify five key clinical predictors of osteoporosis (OP) risk from an initial set of 42 variables: age, sex, body mass index (BMI), uric acid (UA), and alkaline phosphatase (ALP). Among these, BMI exhibited an inverse association with OP risk, consistent with prior findings (23). A study utilizing data from the Taiwan Biobank reported a significant negative correlation between BMI and OP prevalence, suggesting a higher risk among individuals with lower BMI (24). Another analysis proposed that maintaining a BMI between 23.0 and 24.9 kg/m^2^ minimizes the combined risk of OP and type 2 diabetes (25). There exists a complex and bidirectional biological relationship between BMI and osteoporosis. A low BMI markedly increases the risk of decreased bone mineral density (BMD) and fractures by reducing mechanical loading, lowering peak bone mass, and reflecting potential malnutrition or insufficient muscle mass. Reduced body weight decreases the mechanical stimuli exerted on bone tissue, leading to diminished osteogenic activity, while inadequate energy and protein intake further impairs bone matrix synthesis and repair (26). Obesity exerts a dual effect on bone health. Peripheral adiposity may increase bone mineral density (BMD) in certain skeletal sites by enhancing mechanical loading and producing estrogen-like metabolites. However, excessive body fat—particularly visceral adiposity—induces chronic low-grade inflammation and the secretion of pro-osteoclastic cytokines and adipokines (e.g., IL-6, TNF-*α*, and imbalances in adiponectin/leptin), which are often accompanied by increased bone marrow adiposity. The latter impairs bone formation through space-occupying effects and the release of inhibitory factors that suppress osteogenic differentiation of mesenchymal stem cells, ultimately compromising bone quality and elevating the risk of certain types of fractures (27). Sex was also recognized as a critical predictor, with females exhibiting substantially higher susceptibility to OP-related outcomes, including reduced bone mineral density (BMD) and increased fracture risk, primarily due to lower baseline bone mass and estrogen deficiency following menopause (28). Advancing age further compounds this risk. Studies have shown that women aged over 60 years experience accelerated bone loss and higher fracture rates compared to men (9), with OP prevalence reaching 37.3% in women and 7.5% in men over 50 years of age (29). The sharp decline in estrogen after menopause is a key driver of bone loss in women. Estrogen not only directly promotes bone formation by stimulating osteoblast activity but also inhibits bone resorption by upregulating osteoprotective factors such as osteoprotegerin (OPG) and suppressing pro-resorptive signals including RANKL and pro-inflammatory cytokines (e.g., TNF-*α*, IL-1, IL-6). Estrogen deficiency disrupts this balance, leading to increased osteoclast activity and an imbalance in bone remodeling (30). Beyond hormonal influences, sex differences are also reflected in immune and metabolic pathways. Following the loss of estrogen regulation, women are more prone to developing a state of chronic low-grade inflammation and a shift toward pro-resorptive immune cell phenotypes—such as increased Th17 and pro-inflammatory memory T cells. These immune alterations elevate both local and systemic levels of RANKL and inflammatory cytokines, thereby further promoting bone resorption (31). In addition, sex-related factors such as the lineage shift of bone marrow stromal cells (with mesenchymal stem cells more prone to adipogenic differentiation), interactions between the gut microbiota and estrogen metabolism, and sex chromosome– or receptor–dependent differences in gene expression collectively influence osteoblast and osteoclast function as well as the bone microenvironment. These factors jointly contribute to women’s greater susceptibility to bone quality deterioration in response to estrogen deficiency or age-related stress (32). A recent epidemiological study conducted in China in 2023 further supported this trend, reporting OP prevalence rates of 10.7% in men and 51.6% in women aged ≥60 years, thereby underscoring the significant influence of age and sex on OP risk (33, 34). With advancing age, bone tissue undergoes a series of cellular and molecular alterations that lead to reduced bone formation and relatively increased bone resorption, thereby elevating the risk of osteoporosis. Cellular senescence accumulates in bone marrow stromal cells, osteoblasts, and other bone-associated cells, and the resulting secretion of pro-inflammatory and matrix-degrading senescence-associated secretory phenotype (SASP) factors suppresses osteogenic differentiation while enhancing osteoclast activity, directly disrupting bone homeostasis (35). Age-related chronic low-grade inflammation and dysregulation of the immune–bone interplay lead to the upregulation of pro-resorptive cytokines such as RANKL, TNF-*α*, and IL-6, which further accelerate bone loss (31). The increased propensity of bone marrow mesenchymal stem cells to differentiate toward the adipogenic lineage leads to elevated marrow fat accumulation. This not only depletes the cellular pool available for osteogenesis but also suppresses bone formation and disrupts the bone microenvironment through adipose-derived secretory factors, ultimately reducing bone mineral density and bone quality (36). In addition, mitochondrial dysfunction, oxidative stress, epigenetic alterations, and the age-related decline of endocrine factors such as estrogen collectively impair the metabolic and reparative capacity of osteoblasts, reducing bone regeneration and mechanical adaptability. These cumulative effects establish aging as an independent and critical risk factor for osteoporosis (37). The relationship between UA and OP remains controversial. An analysis of the NHANES dataset identified a significant U-shaped relationship between UA levels and OP (non-linear *p* = 0.0287), with BMI fully mediating this association (mediation effect: −0.0024; 95% CI: −0.0026 to −0.0021), highlighting the critical role of body weight in modulating this interaction (38). A separate study involving 13,112 adults from the United States and China revealed a threshold effect between UA and BMD, with BMI accounting for approximately 13.6% of the mediation effect (39). In a cohort of 1,249 OP patients, a non-linear relationship between UA and BMD was again observed, particularly in individuals with BMI < 24 kg/m^2^. Notably, a 100 μmol/L increase in UA concentration corresponded to a 28.6% improvement in BMD, suggesting a potential protective role of moderate UA levels under specific conditions (40). Within a certain physiological range, serum uric acid (UA) can scavenge free radicals and alleviate oxidative stress, thereby preserving osteoblast function and preventing excessive osteoclast activation. This antioxidative property may help maintain bone mineral density (BMD), providing a biological basis for the positive associations observed in several epidemiological studies between low-to-moderate UA levels and higher BMD or reduced fracture risk (41). When UA levels rise to pathological ranges (hyperuricemia) or are accompanied by metabolic disturbances, the resulting excess reactive oxygen species and activation of inflammatory pathways—such as NLRP3 inflammasome activation and upregulation of pro-inflammatory cytokines—can enhance pro-resorptive signaling and disrupt the bone microenvironment, thereby increasing bone loss and fracture risk. Moreover, hyperuricemia is often associated with insulin resistance, endothelial dysfunction, and renal impairment, all of which can further affect bone metabolism by altering bone perfusion, nutrient supply, and endocrine signaling, ultimately shifting the impact of UA on bone from protective to detrimental (42). Therefore, clinical and epidemiological studies often reveal a nonlinear or “U-shaped” association—UA appears to exert a protective effect on bone at low-to-moderate levels, whereas elevated levels or specific metabolic contexts confer detrimental effects (43). Alkaline phosphatase (ALP), a key enzyme in bone metabolism, was also identified as a significant predictor. A study using NHANES data (2005–2018) involving 13,724 adults demonstrated a strong inverse association between total ALP (T-ALP) levels and BMD at both the lumbar spine and femoral neck. Each standard deviation increase in T-ALP corresponded to a 0.5% increase in OP risk (OR = 1.005, *p* < 0.001), and elevated T-ALP levels were associated with higher all-cause mortality among OP patients (44). Similarly, a cross-sectional analysis of 7,796 adults aged 20–59 years revealed a negative correlation between ALP and pelvic BMD (*β* ≈ −0.0008, *p* < 0.000001), with a plateau in BMD decline observed beyond ALP levels of approximately 97 U/L. This non-linear trend was consistent across age, sex, and ethnic subgroups, indicating that ALP may serve as a valuable biomarker for early identification of abnormal bone metabolism and elevated OP risk (45). Alkaline phosphatase (ALP), primarily produced by osteoblasts as bone-specific ALP (BsALP), promotes hydroxyapatite formation by hydrolyzing pyrophosphate, an inhibitor of mineralization. Thus, its serum level reflects bone formation activity and the mineralization process. Elevated or abnormal ALP levels often indicate increased bone remodeling or mineralization disorders, which are particularly evident in high-turnover bone diseases such as Paget’s disease or states of heightened bone metabolism (46). It is also important to note the multisource nature of total ALP. Tissues such as the liver, intestine, and placenta contribute to overall ALP activity; therefore, in assessing bone metabolism and osteoporosis risk, measuring bone-specific ALP (BsALP) provides greater specificity and interpretive value than total ALP (46). Therefore, the observed elevation of ALP associated with increased osteoporosis risk in this study may be interpreted as a biomarker of bone metabolic imbalance, characterized by high-turnover or aberrant mineralization processes (45). Consistent with the SHAP analysis, BMI showed the highest contribution (BMI > Sex > Age > UA > ALP), indicating that the model primarily relied on BMI-related variations to differentiate osteoporosis risk. The model inferred that lower BMI values strongly increased predicted risk, reflecting insufficient mechanical loading and poor nutritional or muscular support for bone formation. Sex and age exerted nonlinear SHAP effects, with postmenopausal females and older individuals showing higher risk contributions. Uric acid displayed a biphasic pattern, suggesting that moderate levels were protective while extremes elevated risk. ALP contributed modestly, indicating its role as a secondary marker of bone turnover. The SHAP pattern reveals that the model prioritized variables capturing metabolic, hormonal, and structural determinants of bone fragility. Collectively, the five variables identified via LASSO regression—BMI, sex, age, UA, and ALP—represent clinically meaningful and evidence-supported predictors of OP, forming a robust foundation for risk stratification and personalized screening.

SHapley Additive exPlanations (SHAP) were incorporated to interpret the Random Forest model developed for osteoporosis (OP) risk prediction. The global SHAP importance ranking indicated that BMI was the most influential predictor, as evidenced by the highest mean SHAP value, followed by sex, age, uric acid (UA), and alkaline phosphatase (ALP). At the local interpretation level, SHAP force plots clearly illustrated both the direction and magnitude of each feature’s influence on OP risk prediction for individual patients. Higher BMI and elevated UA levels were associated with negative contributions—suggesting protective effects—while increased age and elevated ALP levels were linked to positive contributions, reflecting their role in the pathogenesis of OP. The clinical utility of SHAP in disease prediction has been supported by prior studies. A comparative evaluation conducted by Elias et al. highlighted that SHAP significantly enhances interpretability and trust in clinical models, enabling the identification of dominant risk features for personalized intervention (13). Rietz et al. further demonstrated the integration of SHAP into the FREM-ML fracture risk model within clinical workflows, emphasizing the importance of visual explanations of individual-level risk drivers to facilitate treatment decisions (47). Similarly, Ghasemi et al. reported that SHAP, as a widely adopted model-agnostic interpretability method, substantially enhances transparency and credibility in oncology diagnostic modeling, and has shown robust generalizability across medical AI applications (18).

In this study, the Random Forest-based model was implemented as a web-based visualization tool to improve clinical applicability. By inputting five key variables—age, sex, BMI, UA, and ALP—the tool provides real-time individualized predictions of OP risk, accompanied by SHAP force plots that visually quantify each feature’s contribution. This enhances both interpretability and clinical usability in decision-making. In recent years, the integration of interpretable and visualized machine learning models into clinical workflows has gained traction. Lai et al. reported in a multi-cohort validation study that encapsulating complex machine learning models into interactive web applications can transform model predictions into intuitive risk assessment tools. Such applications also enable visualization of feature importance, thereby mitigating the “black box” criticism and enhancing clinical interpretability and acceptability (48). Lin et al. also proposed a web-based clinical decision support system (Web-CDSS) for AI-assisted decision-making, in which the predictive modeling and visualization interpretation modules (similar to SHAP outputs) are tightly integrated to facilitate interactive use by clinical practitioners (49). Yang et al. developed an interpretable machine learning model for low bone density risk assessment and subsequently deployed it as a web-based platform to provide clinicians with real-time risk prediction and model interpretability services (50). These studies demonstrate that integrating predictive models with web-based visualization interfaces enables clinicians without technical backgrounds to intuitively observe predicted probabilities as well as the direction and magnitude of key variable contributions to risk, thereby enhancing the usability and trustworthiness of such tools.

This study has several limitations. First, the data were obtained from a single medical institution, and the regional specificity of the sample may limit the model’s generalizability, making its applicability in different healthcare settings less robust. Second, all biochemical indicators were measured in a single laboratory, which may introduce measurement bias and affect variable consistency. Third, this study employed a retrospective design without any form of external validation (e.g., temporal, geographical, or inter-institutional validation). The absence of performance and calibration assessment using independent external cohorts may lead to an overestimation of the model’s true transferability. In addition, FRAX and GARVAN scores were not available in our dataset, which prevented comparison with established clinical risk assessment tools. Future studies should expand the sample size and incorporate multicenter and multi-regional data to strengthen external validation and enhance clinical applicability. We plan to conduct independent validations across different time frames and healthcare institutions or regions, systematically reporting model discrimination (AUC/AUCPR), calibration (Brier score, calibration slope/intercept), and clinical net benefit (decision curve analysis, DCA).

## 5 Conclusion

Based on a large-scale clinical dataset, this study identified five key predictive variables—age, sex, BMI, UA, and ALP—using LASSO regression and compared multiple machine learning algorithms. The Random Forest model was ultimately selected as the optimal predictive approach, achieving an AUC of 0.759 in the independent test set, indicating moderate predictive performance. Model interpretability analysis using SHAP clarified the relative contribution of each variable to osteoporosis risk prediction. Furthermore, a web-based visualization tool was developed based on this model to enable automated, individualized assessment of osteoporosis risk. External validation and recalibration using multicenter datasets are still required to confirm the model’s robustness and clinical applicability.

## Data Availability

The datasets presented in this study can be found in online repositories. The names of the repository/repositories and accession number(s) can be found in the article/supplementary material.
